# Phased Introduction of Haemodialysis in Patients with Kidney Failure: A Mixed-Methods Feasibility Study

**DOI:** 10.3390/healthcare14060792

**Published:** 2026-03-20

**Authors:** Adil M. Hazara, Maureen Twiddy, Victoria Allgar, Sunil Bhandari

**Affiliations:** 1 Department of Renal Medicine, York Hospital, Wigginton Road, Clifton, York YO31 8HE, UK; 2 Hull York Medical School, University of York, York YO10 5DD, UK; sunil.bhandari@nhs.net; 3 Institute of Clinical and Applied Health Research, University of Hull, Hull HU6 7RX, UK; maureen.twiddy@hyms.ac.uk; 4 Peninsula Clinical Trials Unit, Faculty of Health, University of Plymouth, Plymouth Science Park, Plymouth PL6 8BX, UK; victoria.allgar@plymouth.ac.uk; 5 Academic Renal Research, Hull University Teaching Hospitals NHS Trust, Hull HU3 2JZ, UK

**Keywords:** phased haemodialysis start, kidney failure, dialysis treatment, feasibility study, incremental HD

## Abstract

**Background**: Introducing haemodialysis (HD) treatment in a phased manner, with lower treatment times at the outset combined with pre-defined increments in treatment over a period of several weeks, reduces the early burden of treatment in patients with kidney failure and may help improve early outcomes. We have evaluated the feasibility of a novel transitional HD regime using a mixed-methods approach. **Method**: A single-centre cohort design was adopted, where participants were enrolled prospectively into an interventional arm and matched with historical controls. This paper reports on the feasibility of recruitment and retention in the prospective arm. People with kidney failure, starting HD treatment in out-patient settings, were recruited. They started HD on a transitional regime, with four pre-specified incremental steps (Phases 1 to 4), which aimed to establish participants on long-term 3× weekly treatments over 14 weeks. Participants’ experiences of starting HD in a phased manner were analysed using semi-structured interviews. **Results**: We screened 127 people over 18 months: eligible: 54 (43%); enrolled: 25 (46% of eligible). Fifteen started HD within the study timeframe; 14 were retained for 6 months. In 13 participants, the regime was altered (mostly during Phase 2) for clinical or scheduling reasons. Semi-structured interviews (*n* = 11) found participants overwhelmingly liked the phased HD introduction as an aid to becoming normalised to dialysis routines. Alterations to treatment were not associated with adverse experiences. Participants would highly recommend starting dialysis in this stepped and phased manner. **Conclusions**: It is feasible to enrol and retain participants in the proposed program of phased start of HD. The regime may be implemented flexibly in future trials. Starting dialysis on a less-than-three-times weekly basis was well received by participants. **Trial Registration**: Clinicaltrials.gov registration NCT04268264 (registered: 11 February 2020).

## 1. Background

Starting haemodialysis (HD) therapy at a reduced frequency, with the aim of establishing patients on long-term treatment, may help reduce some of the early risks faced by people with kidney failure. Reducing the treatment burden when patients first transition into dialysis dependency could help them adapt more easily to the physical and mental challenges of long-term dialysis [1,2]. An *incremental* start, as an alternative to the conventional three-times weekly treatments, may be considered optimal [3] in patients who still have a significant residual kidney function [4,5,6], though there have been significant challenges in its widespread adoption and implementation.

Observational studies comparing outcomes between twice-weekly programs of HD and conventional start [7,8,9,10,11,12] show no signals for harm when dialysis with reduced starting frequency was administered alongside close patient monitoring, and when incremental steps were pre-defined and pre-planned. Ultimately, a fully powered randomised controlled trial (RCT) of infrequent vs. conventional start will be needed to definitively explore if this strategy improves early survival in incident people receiving dialysis treatment [13,14].

A rigorous approach is essential in the refining and testing of new programs that start patients on twice-weekly HD treatment at the outset, to prepare these for fully powered RCT in the future, and to help them meet the challenges of implementation in clinical practice. We conducted a mixed-methods feasibility study to test the acceptability of, and adherence to, a novel program of stepped and phased introduction of haemodialysis in people with kidney failure. Our objectives were also to explore patients’ experiences, using semi-structured interviews, of starting HD using this approach and participating in the current study.

## 2. Methods

### 2.1. Settings

The study was based at a large teaching hospital in the UK and its associated satellite dialysis units. This centre provides tertiary care renal services to a catchment area population of 1.25 million in the North East of England [15].

### 2.2. Participants and Study Design

Full methods for the current study have been published previously [16]. A cohort study design was adopted. Adults commencing in-centre maintenance HD therapy for established kidney failure (from any cause) in the out-patient settings were enrolled over an 18-month period and followed up for up to six months. Full eligibility criteria are presented in Supplementary Table S1. These participants were matched with historical controls (1:2 ratio), i.e., people who had previously started dialysis treatment on a 3× weekly basis at our centre since 2013 (date of inception of our modern dialysis database). Hence, the study comprised an active interventional arm recruited prospectively and historical controls representing the conventional therapy arm. We have previously published the rationale for this approach [16]. This paper reports on the feasibility of recruitment and retention in the prospective arm and analyses of participant interviews (secondary outcomes comparing in detail the indicators of safety and wellbeing are also undergoing peer-review at this journal as a separate paper).

### 2.3. Recruitment Strategy

Potential participants were identified from out-patient clinic lists and through the referral pathway for new dialysis starters. Participants’ interest in the study was first gauged by their own clinical teams, who also provided the participant information sheet. The investigator (first author) then approached those interested in the study for full counselling. They were given at least 24 h to consider all information. Recruitment to the prospective arm was completed over two periods interrupted by the first wave of the COVID-19 pandemic. Between May 2019 and March 2020 (11 months), 21 participants were enrolled; then, following a pause in recruitment, four further participants were added between September 2020 and March 2021 (7 months). These participants remained inactive in the study, and under follow-up with their usual kidney specialist, until the start of HD. The timing of HD initiation was made independently of the study investigators by patients’ own kidney physicians, based on clinical needs and in consultation with patients.

### 2.4. Sample Size

As this was a feasibility study, a formal sample size calculation was not performed. In the pre-coronavirus disease-2019 (COVID-19) pandemic protocol, a pragmatic recruitment target of 40 participants in the interventional arm was set. We expected 85 new dialysis starters over the recruitment period based on renal registry data [17] and aimed to recruit 40 of them. The target had to be revised down to 20 due to the pandemic. Hence, recruitment to the study was severely impacted by the events of the pandemic.

### 2.5. The Intervention: The Stepped and Phased Regime of Incremental Hd

**Figure 1:**
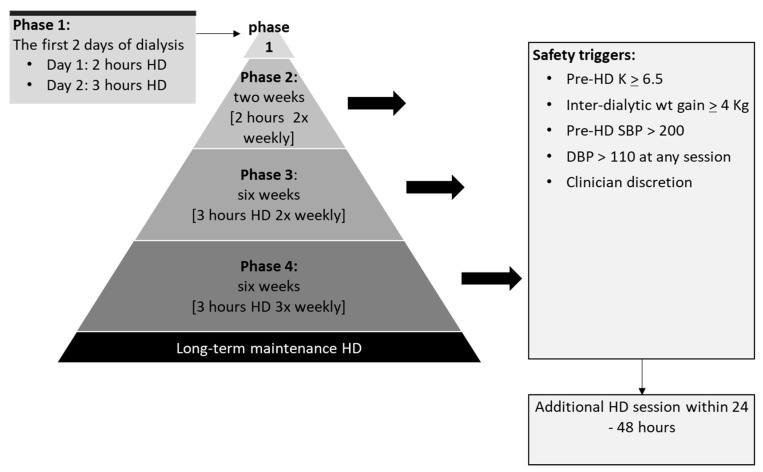
The program of phased introduction of haemodialysis used in this study, showing the incremental steps. SBP = systolic blood pressure; DBP = diastolic blood pressure in mmHg; K = potassium in mmol/L (modified from Hazara et al., 2021 [16]).

Participants in the intervention arm started therapy on a 2× weekly basis with progressive and pre-planned increases in the duration and frequency of sessions over 14 ½ weeks (14 weeks and 2 days), achieving conventional treatment times by the end of this period (see Figure 1). The key features of this regime are: (a) it applies to people who are new to dialysis and are starting dialysis in a pre-planned manner in the out-patients setting; (b) the dialysis treatments start with reduced frequency and times; (c) there are provisions for regular monitoring of key parameters of patient wellbeing through regular blood checks, fluid balances assessments and clinical reviews; and (d) the regime has pre-defined steps for increments in dialysis treatment and includes safety triggers that would result in an increase in dialysis to match patients’ needs; see our previous publication for full details [16]. Oral medications such as diuretics and sodium bicarbonate were continued.

### 2.6. The Interviews

All participants in the intervention arm (i.e., those who had started HD using the stepped and phased approach described above) were approached. Interviews were conducted over the telephone by the first author using Cisco Jabber version 12.5 (Cisco Systems Inc., San Jose, CA, USA). All interviews were recorded with participants’ consent. Telephone interviews were used to minimise patient exposure to COVID-19 and were carried out when patients were at home (i.e., away from the dialysis unit) to ensure their privacy. The topic guide used to capture patients’ experiences of receiving the intervention and participation in the feasibility study has previously been published [16] and is reproduced in Table 1.

### 2.7. Outcome Measures

The pre-specified feasibility outcomes were defined such that feasibility would be demonstrated if 40% of people approached for participation consented to the study (acceptance) and 75% completed the program as planned at the start (adherence). Exploratory outcomes included documenting completion rates of non-routine tests administered as part of the study, namely: the KDQOL-SF v1.3 questionnaire [18], urine collections, and bioimpedance measurements. The quantitative findings are supported by interview data to build a more complete picture of factors that may have influenced acceptance, adherence, and patients’ completion of additional tests. This paper focuses on feasibility and qualitative outcomes; comparisons of clinical indicators of patient safety and wellbeing between treatment groups are being published separately.

### 2.8. Analysis

The findings of this study are reported in accordance with the CONSORT Statement for Pilot and Feasibility Trials [19]. We present the proportion of people screened, approached and recruited; the statistics presented are descriptive. Deviations from the planned incremental regime are presented per participant in a summary table. The interviews were transcribed verbatim and managed using NVivo 13 (QSR International, Melbourne, Australia). The typed transcripts were pseudonymised. Thematic analysis [20] was undertaken to identify and interpret patterns of meaning, i.e., ‘themes’ [21] from the data, with codes and themes discussed with author 2 (MT).

## 3. Results

### 3.1. Quantitative Phase

#### 3.1.1. Recruitment

**Figure 2:**
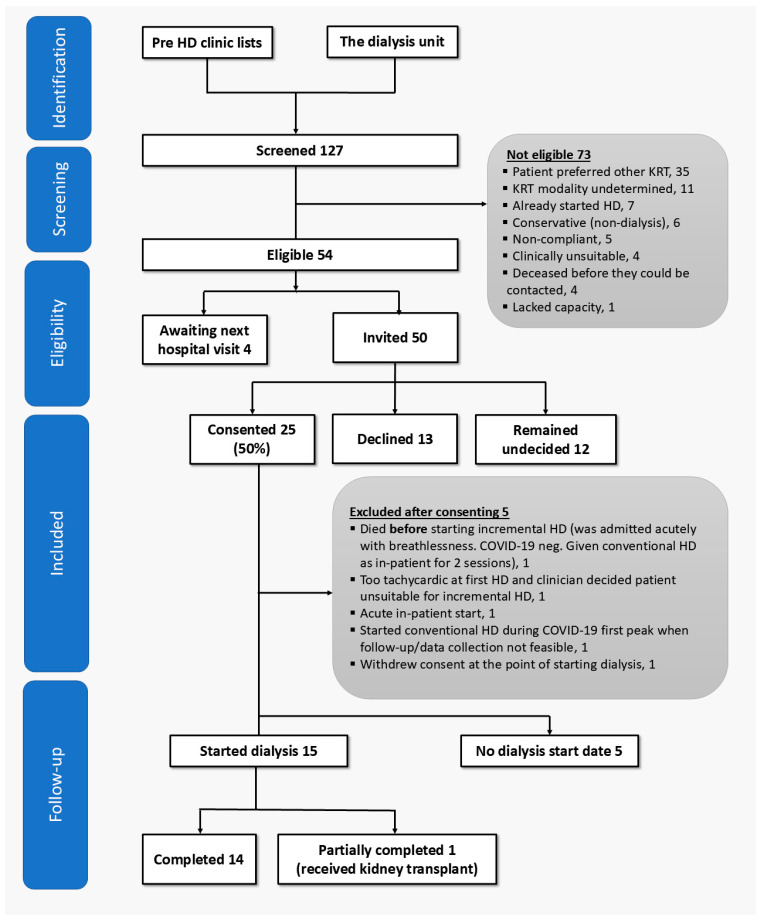
CONSORT flow chart. (KRT: kidney replacement therapy; HD: haemodialysis; COVID-19: coronavirus disease-2019).

Over 18 months, 127 people were screened for participation in the experimental arm (see Figure 2). Of these, 54 (43%) were eligible. Reasons for ineligibility are shown in Figure 2. Of the 54 eligible people, 25 (46%) consented to the study, hence meeting the pre-defined feasibility threshold (of >40%) for recruitment. The screen-to-consent proportion was 25/127 (20%). The overall enrolment rate was 1.4 participants/month. The 25 people who consented were similar in age (65.3 years vs. 64.6 years) and proportion of males (16/25 [64.0%] vs. 122/191 [63.9%]) compared to all new planned out-patient HD starters since 2014 (*n* = 191) at the host institution.

Fifteen of the 25 participants (60%) eventually started incremental HD during the study period. Median time from enrolment to commencement of dialysis for participants recruited from the pre-dialysis clinic was 113 days (range: 8–361 days). Of the 10 participants who failed to progress in the study beyond consenting, five did not require dialysis during the study, whilst the other five were excluded for reasons shown in Figure 2.

#### 3.1.2. Adherence

Changes made to the treatment during each phase of the study per participant are summarised in Table 2. Two of the 15 participants received treatment entirely as planned during all phases of the incremental HD regime. In six of the 15 participants, relatively minor changes were needed in the program; these were due to: extravasation of blood from a displaced dialysis needle, requiring resting of the fistula (two participants); interruptions in the scheduling of dialysis during national holidays (two participants); poorly matured fistula at the outset, causing needling difficulties and extension of treatment times (one participant); and missed dialysis session due to patient concordance (one participant).

Treatment was escalated to the next incremental step ahead of schedule (usually Phases 2 to 3) in five of the 15 participants. Reasons for these increments were the need for additional ultrafiltration as assessed clinically (two participants), persistent hyperkalaemia of ≥5.5 mmol/L (one participant), anaemia compounded by concerns about patient concordance (one participant) and needling difficulties at the fistula compounded by persistent hypertension (one participant). In two participants, disruptions caused by hospitalisations resulted in changes to treatment times.

Changes to planned treatment were most common during weeks 2 and 3 (Phase 2) of the incremental regime. Of the 15 participants, adjustments were made to treatment in nine (60%) participants during Phase 2. By contrast, only three (20%) participants had changes made to their treatment during weeks 4 to 9 (Phase 3) and two (13%) during weeks 10 to 15 (Phase 4).

#### 3.1.3. Completion of Non-Routine Tests

Out of a total of 58 possible urine collections, 51 (88%) were completed. Completion rates were 93%, 80%, 86% and 93% at baseline, one month, three months and six months respectively. Mean urine volume at baseline was 1308 (95% CI 1153–1464) mL per 24 h. All participants had urine volume > 400 mL/24 h. The mean change in urine volume from baseline was −80 (95% CI −303 to 141) mL/24 h, −370 (95% CI −604 to −135) mL/24 h and −671 (95% CI −900 to −443) mL/24 h at one, three and six months respectively.

Over-hydration was measured using multifrequency bioimpedance. A total of 88 measurements were planned in the entire cohort. The actual number of readings taken was 76—completion rate: 86%. Over-hydration was estimated at 1.8 (95% CI 1.0 to 2.5) L at baseline. The mean change in over-hydration was 0.3 (95% CI −0.8 to 1.3) L, −0.1 (95% CI −1.1 to 1.0) L and −1.0 (95% CI −2.1 to 0.0) L at one, three and six months respectively.

In total, 43 KDQOL-SF questionnaires [18] were given out: 15 at baseline, 14 at three months and 14 at six months. The overall response rate was 77% (33 returned questionnaires). The return rate was 12/15 (80%), 9/14 (64%) and 12/14 (86%) at baseline, three months and six months respectively.

### 3.2. Patient Experiences of the Trial

Interviews lasted for up to an hour. Analysis of the interviews identified two main themes: (1) ‘Preparing for and starting dialysis’ and (2) ‘Experience of participating in the study’. The first theme describes participants’ experiences just prior to starting treatment, during which time they were under the care of specialist kidney teams, and then their experiences after the onset of dialysis treatment. The second theme describes the participants’ views of participating in this study.

#### 3.2.1. Theme 1: Preparing for and Starting Dialysis

##### Prior Knowledge and Preparation for Dialysis

For most patients, knowledge about dialysis came from clinicians, with a few searching the internet. However, participants expressed a sense of inadequate preparedness for dialysis and a fear of the unknown. There was a feeling that the information delivered to them was too generic and not personalised to their needs: “no one really explained what it would mean for me”—SF7.

Perception of negative effects that dialysis would bring to participants’ lifestyles was quite common; life on dialysis was perceived as being “grim” (HU1), almost akin to entering the early phase of dying. It was perceived to be something which must be avoided, or at least delayed, as much as possible. These perceptions were re-enforced by the participants’ own observations of other people receiving dialysis during clinic visits and pre-dialysis counselling: “(the dialysis unit) just looked to be full of people that obviously seemed quite ill and were going through this ordeal of having dialysis”—HU1.

##### Acceptance and Overcoming Fears

Despite these concerns, participants were stoic about starting dialysis, accepting of what was being offered to them: “but I thought well I have been through a lot of stuff. I can get through it”—NN14. Despite the compromises they faced, participants saw the start of dialysis as an existential necessity. Many felt that they had little choice but to accept medical advice and fit into the established routines: “(the nurse) says, you know, this is what’s expected and I went fine”—LC17.

##### Resolution of Symptoms of Late CKD

With the onset of dialysis, some of the symptoms usually associated with the retention of uraemic toxins started receding. The return of appetite, and the resolution of nausea, itching and symptoms related to gout were reported early on. When accounting for changes in their health after the onset of dialysis, participants attributed changes to alterations in treatment frequency rather than session length (duration) or any other aspect of treatment: “you’re feeling a lot better when you are on three times weekly than you are on twice weekly…. It must have been my changes in the blood you felt better”—KI23.

#### 3.2.2. Theme 2: The Experience of Participating in the Study

##### Adequacy of Information

Although participants felt well informed about the study and the procedures it entailed as their dialysis treatment progressed, participants sought information about how they were ‘getting on’ on dialysis and, in particular, what their test results were. Participants found the study-related tests “no trouble at all” (NN14), interpreting the frequent testing as being the same as close monitoring. However, these additional tests also heightened participant interest in learning the test results: “A little more information would be quite useful. I don’t wanna become a hypochondriac but I like to know what’s going on and don’t get enough chat really”—UU22.

##### Incremental HD as ‘Easing Into’ Dialysis

Participants bought into the idea that being gradually introduced to dialysis treatment was beneficial as it “eased” (NN14) them into the treatment routines. There was widespread acceptance of the inevitability of eventually receiving three times weekly treatments, yet the less frequent start enabled them to “acclimatise” (UU22) to living on dialysis long term: “the idea of introducing people into it slowly, I felt was a good idea and it’s something that I’ve really appreciated”—HU1.

Dialysis was experienced as a disruption to normal life, and there was a fear of going on dialysis. Starting dialysis was perceived by participants to be quite heavy-handed, with descriptions such as “brutal” [WK11], “being thrown” [LC17] onto the treatment used to describe dialysis treatment and coming to harm from side-effects of the treatment. In contrast, incremental dialysis was seen as easing the “trauma” (UP25) associated with the starting dialysis, and as a way of minimising the proverbial “shock to the system” (NN14; LC17).

This need for acclimatisation to dialysis was about more than just meeting the physical demands of dialysis; it was also the mental health aspect of starting the treatment that was important to the participants.

“I think I might have suffered with a degree of depression to be honest”—HU1.

“I think I would have ended up in a depression if I went on it straight away, for four hours”—LC17.

They saw the start of dialysis as part of a whole package of changes to their lives.

“you’ve gotta get used to the nurse putting the needles in you know”—KI23.

“by doing gradually getting to know the staff that are in your area looking after you”—WK11. Incremental dialysis allowed participants to overcome their anxieties related to their new social surroundings more gradually.

Participants cited their worries about maintaining their job—“I still want to carry on working, like to have a normal life”—LC17—and enjoying their retirement as further reasons for agreeing to start dialysis incrementally. Participants also perceived it to have spared them some of the treatment-related side effects early on: “I think if you went on it straight to four hours, you know what I mean, I think you would feel, it is a shock to the body and I think you would feel drained, poorly”—LC6. Some participants said they would have liked to continue on twice-weekly treatments if this were an option: “If I could get away with only two hours all the time I’d be glad”—LC17. In contrast, others said they felt better on full three-times weekly treatments, so the benefits of twice weekly were relatively short-lived and related more to psychological and emotional adjustment: “you felt a little better when you’re on 3 h [i.e., later in the program]”—KI23.

##### Altruistic Reasons and for Societal Good

Prominent among the reasons for patients wanting to participate in this study was the desire to help. LC17, who worked for the NHS herself, realised the necessity of studies such as this, which aim to help patients with long-term health conditions; similar feelings were expressed by other participants who wanted to contribute to research that would eventually help patients in the future.

## 4. Discussion

The current study shows that it is feasible to proceed with a program of phased and stepped introduction of HD in people with kidney failure, albeit with modifications (i.e., the omission of Phase 2 of the program—see Figure 1). The study met its pre-defined threshold for acceptance (46% of people eligible for participation consented). Strict adherence to the pre-specified incremental steps was low, though measures put in place and offered as part of the program enabled modification and tailoring of the treatments based on clinical needs. Further, the interview data revealed that the current program of phased introduction of dialysis was very popular among study participants who expressed a high degree of support for the idea of incremental start.

The importance of involving patients in making shared decisions regarding the management of CKD is emphasised in national guidelines [22]. Whilst clinicians may focus more on biomedical outcomes in the management of advanced CKD, patients and their carers are likely to place higher value on lifestyle outcomes [23]. Qualitative research methods give voice to people and provide detailed descriptions of experiences [24]. They have an increasingly prominent role in process evaluation works and on the utility of interventions from the recipients’ perspective [25,26]. This study employed both quantitative and qualitative analyses to provide a more in-depth assessment of feasibility than the traditional ‘stop/go’ [27] criteria for progression. The presentation of patients’ perspectives, and their role in evaluating the proposed intervention, is innovative in incremental HD research; it helps make the programme more relevant to patients’ needs and prepares it well for future applications to regulators and funders.

Participation in this study was limited to those starting dialysis in a planned manner and exclusively from out-patient settings. People starting dialysis acutely as in-patients were excluded. This approach yielded a cohort of participants with a good level of residual kidney function at baseline. The strategy adopted here, therefore, represents a useful approach to recruitment in a future trial and is associated with positive participant experiences. The time from enrolment to participants commencing dialysis varied between 8 and 361 days (median 113 days). This reflects differences in the rate of progression of CKD, patient preferences and logistical factors. Recruitment strategy in a full-scale clinical trial in the future must account for this by approaching potential participants early with an acknowledgement that a proportion of participants may not start dialysis within the trial period.

The proportion of participants who received the intervention entirely as planned was low. Although some of the changes made to treatment times were predictable, e.g., scheduling issues during the holiday period, other changes that were made reflected acute clinical problems, which were not predictable at the start, such as hyperkalaemia and fluid overload (in a minority of patients). The protocol, however, did entail in-built safety measures, and decisions to deviate from prescribed treatment times were made by clinicians in a proactive manner based on changing clinical needs. The approach taken is in keeping with the ethos of making dialysis trials more reflective of real-world practice [28] and prioritises individualised treatments over the adoption of a rigid approach in delivering dialysis, as advocated by the participants in the Kidney Disease: Improving Global Outcomes controversies conference related to dialysis practices [29].

When deviations from set treatment times were examined per phase of the incremental regime (see Figure 1), the two-week-long 2 h/week schedule (Phase 2) seemed most problematic; 60% of all deviations occurred during this phase. Phase 2 represented a minimal period of dialysis purely to help familiarise patients with the dialysis routines. However, such short treatment times leave little margin for error or time for dealing with any unexpected complications, such as the dialysis needles becoming dislodged, which would require prolongation of treatment times and/or rescheduling of treatment. In future versions of this regime, the inclusion of Phase 2 appears unfeasible. A modified regime, for implementation in a future clinical trial, would simply start with Phase 3 (three-hour, twice-weekly sessions; see Figure 1), followed by Phase 4 for a further 8 weeks. This is likely to result in fewer additional clinical interventions and better resource utilisation.

Despite the changes made, participants were accepting of the overall regime and were happy to go along with treatment recommendations; it did not seem to negatively impact their experiences of starting dialysis incrementally. Hence, in future studies, a less rigid approach may be more appropriate when evaluating ‘adherence’, which should be seen as fidelity to protocol with a pragmatic delivery model. It is acknowledged, however, that providing HD flexibly may not always be possible within the current model of care, where dialysis provision is mostly centred around catering for three-times weekly 4 h sessions.

Participant retention in the prospective arm was excellent (14 of 15 participants completed the programme). Note that retention was defined as the proportion of participants who actively contributed to study procedures and data collection throughout the six-month follow-up period. Participants expressed a high level of satisfaction with study procedures and felt well looked after. They were overwhelmingly positive about the merits of starting dialysis incrementally and liked their interaction with the study personnel. Their positive experiences may be the reason behind participants’ active engagement in data collection activities in this study and the high completion rates for non-routine tests.

Although this study reports on the feasibility of recruitment, retention, and adherence to our proposed method of initiating haemodialysis, we anticipate that this regimen may be most suitable for patients starting dialysis in a planned out-patient setting. These patients are typically well prepared for the initiation of dialysis, having received appropriate education and training and having established vascular access. They are also more likely to be engaged and activated in their self-care. Potential barriers to implementation include the need to overcome the traditionally rigid structure of thrice-weekly dialysis scheduling, the requirement for additional staff training, and the risk of service disruption during holiday periods. In a future clinical trial, early problems common among new haemodialysis starters, such as difficulty cannulating a new arteriovenous fistula, could be anticipated and mitigated through structured protocols for access management [30], although this may have resource implications.

This study has several limitations. Due to the impact of the COVID-19 pandemic, the recruitment target was not met. Note, however, that the feasibility outcomes were all exploratory; it is acknowledged, though, that a greater number of participants may have helped strengthen the findings presented. Additional participants would also have added further perspectives during the interview phase of the study, maximising variations in expressed views and enriching the available data further. This study did not randomise patients, and it remains unclear if participants would have agreed to the study had there been a 50/50 chance of receiving incremental HD. The recruitment and retention rates reflect interest in the twice-weekly HD start programme itself. This, however, can be addressed in a future RCT with an adaptive design, the first phase of the RCT being a study of the feasibility of randomisation.

## 5. Conclusions

The quantitative and qualitative findings combined may be seen as evidence of high interest in reduced-frequency dialysis when patients first transition into dialysis dependency. Our findings support the pursuit of the current approach of starting HD on less than a three-times weekly basis and set the stage well for future research in this area. The research presented here has resulted in the generation of a significant new understanding of the experiences patients go through with the incremental approach to starting HD.

## Figures and Tables

**Table 1:** The interview topic guide (from Hazara et al., 2021 [16]).

Questions	Prompts/Clarifications
Looking back to a time when you hadn’t yet started dialysis (i.e., when you were still under follow-up at pre-dialysis clinic), what were your expectations from dialysis?	Did you have any prior concerns about starting dialysis? What changes were you expecting dialysis would bring to your lifestyle or living arrangements? What changes were you expecting dialysis would bring to your symptoms?
What did you think about being approached for participation in the study?	You were approached in …………. (Clinical setting), by ………… (study personnel), how did you find the experience? Would you suggest we did anything differently when approaching participants for the study in future? What went well? What could be improved?
What were the main reasons you said yes to participation in the study?	Did you discuss the study with your family? Did you discuss the study with other health professionals?
Did you have any concerns at the start about participating in the study?	Do you think your concerns were adequately addressed? In hindsight, what do you think we could have been done differently to pre-empt and address these concerns?
What are the main things that you got from the study?	Do you think there have been any advantages to taking part in the study?
Did you experience any problems?	How did the problem affect you? How did the problem affect your family/carers? In hindsight, what do you think could have been done to avoid this problem?
What changes, if any, do you think we need to make to the dialysis programme (the ‘incremental dialysis’) itself?	In incremental dialysis, you initially receive treatment twice weekly at shorter durations than usual, then your treatment is increased gradually over three months. I would like to get your thoughts on this programme: When you first started dialysis, did you notice an immediate change in how you felt? Were your symptoms changing with increasing dialysis (for better or worse)? You are now on three-times weekly full-length dialysis. With hindsight, did you have any symptoms previously that you think could have been because of less frequent dialysis?
The study included several additional procedures and tests that are not offered routinely to dialysis patients (these included questionnaires and urine collections). How did you find completing those tests?	Anything to report for the 24-h urine collections? Anything to report for the quality-of-life questionnaires? In future studies, should these tests be conducted more often, less often or at the same number of times as in this study?
Would you suggest we did anything differently in this study?	What changes do you think we need to make to the study overall to improve the incremental dialysis regime?
Would you recommend the study to other patients in future?	What advice would you give to someone who is about to start dialysis treatment and is offered a chance to start dialysis incrementally as part of a research study?

**Table 2:** Adherence to the treatment regime. Note that changes to planned treatment were most common during Phase 2 of the incremental regime.

ID	Phase 2 (2 h Twice Weekly Treatments for 2 Weeks)	Phase 3 (3 h Twice Weekly Treatments for 6 Weeks)	Phase 4 (3 h Three-Times Weekly Treatments for 6 Weeks)	After Phase 4
1	Extravasation of blood following needle dislodgment (‘fistula blow’) at the first session; subsequently developed haematoma at needling site. Received 60 min of additional dialysis.	Treated as planned.	Treated as planned.	Received 3× (4 h) weekly HD.
2	Christmas and New Year dialysis scheduling at the satellite unit prevented the administration of 2 h twice weekly treatments with pre-HD potassium checks.	Treated as planned.	Treated as planned.	Received 3× (4 h) weekly HD.
3	Increased to 3 h twice weekly from week 2; pre-dialysis potassium persistently above normal range (peak 5.7 mmol/L).	Dialysis regime interrupted by loss of fistula (thrombosis, collaterals), the requirement for a new line and an associated complication related to line insertion.	Treated as planned.	Received 3× (4 h) weekly HD.
4	60 min additional dialysis in week 2, to enable ultrafiltration to achieve target weight (patient started dialysis fluid overloaded).	Treated as planned.	Treated as planned—except continuation of reduced times during phase 4 over Christmas and New Year.	Received 3× (4 h) weekly HD.
5	In the first week of treatment, fistula ‘blew’ and required resting.	Treated as planned.	Treated as planned.	Received 3× (4 h) weekly HD.
6	Regime interrupted by an episode of hospitalisation for infection of unknown origin (cultures negative) during week 3. Whilst an in-patient, received 3× weekly HD as guided by the attending consultant.	Continued on the 3× weekly 3 h sessions. Hospitalisation for gastrointestinal symptoms in week 7 (2 days). Further hospitalisation in week 8 for pyrexia of unknown origin (6 days).	From week 9 onwards, received 3× (4 h) weekly HD. Note fewer dialysis hours in week 10 due to Christmas/New Year scheduling.	Continued receiving the conventional 3× (4 h) weekly HD. In week 21, patient sustained a non-ST-elevation myocardial infarction.
7	Regime escalated to next phase one week ahead of schedule due to persistent hyperkalaemia (later investigations showed patient also had adrenal sufficiency).	Treated as planned.	Treated as planned.	Clinician decided to maintain participant on the same regime as during phase 4 of incremental HD.
8	Treated as planned.	Treated as planned.	Treated as planned.	Received 3× (4 h) weekly HD.
9	Treated as planned.	Treated as planned.	Treated as planned.	Received 3× (4 h) weekly HD.
10	Treated as planned.	One hour of additional dialysis in week 5 to enable blood transfusion. Fistula needed to rest in week 6 for ‘fistula blow’.	MDT decision to escalate to 3× (4 h) sessions ahead of schedule during week 13. Reasons unclear, but this was during the 1st wave of COVID-19 pandemic (fewer staff available), patient had recently missed sessions due to compliance and had experienced recent fistula needling difficulties.	Continued on regular 3× weekly HD.
11	Treated as planned—participant joined study in week 2 of her dialysis.	Treated as planned.	Missed session during week 10 (patient compliance). From week 12 onwards, developed poor flows in venous limb of dialysis fistula, resulting in a missed session during week 12.	Continued on regular 3× weekly HD, albeit sub-optimally due to persistent line flow problems. Eventually required hospital admission for line infection and change.
12	Started dialysis with extensive fluid overload, hence phase 2 treatment could not be delivered as planned, and participant received 2× (4 h [in week 2] and 3 h [in week 3]) HD sessions to enable additional ultrafiltration.	Treated as planned.	Treated as planned.	Received 3× (4 h) weekly HD.
13	Only single-needle dialysis was possible during week 2 due to needling difficulties; hence, treatment times were temporarily increased to compensate for this less efficient form of dialysis.	Treated as planned.	Treated as planned.	Received 3× (4 h) weekly HD.
14	Poorly matured fistula at the outset resulted in needling difficulties and extension of dialysis time.	Remained on escalated treatment of 2× (4 h) weekly instead of the planned 2× (3 h) weekly treatments due to known access problems, persistent hypertension and raised pre-dialysis K (peak 5.7).	Patient received transplant and remained well in the peri-transplant period.	Continued with functioning kidney transplant
15	Treated as planned—participant joined the study during week 1.	Additional dialysis due to Christmas and New Year holiday period—otherwise treated as planned.	Treated as planned—hospitalisation for COVID-19 (no change needed in treatment regime).	Received 3× (4 h) weekly HD.

## Data Availability

The data presented in this study are available on request from the corresponding author. The data are not publicly available due to privacy and ethical restrictions.

## Supplementary Materials

The following supporting information can be downloaded at https://www.mdpi.com/article/10.3390/healthcare14060792/s1, Table S1: Eligibility criteria for study participants.

## Abbreviations

CKD	Chronic kidney disease
CONSORT	Consolidated Standards of Reporting Trials
COVID	Coronavirus disease 2019
DBP	Diastolic blood pressure
HD	Haemodialysis
KDQOL	Kidney disease quality of life (questionnaire)
KRT	Kidney replacement therapy
MDT	Multi-disciplinary team
NHS	National Health Service
RCT	Randomised controlled trial
SBP	Systolic blood pressure
UK	United Kingdom
USA	United States of America
